# Oncology Nurses’ Attitudes, Knowledge, and Practices in Providing Sexuality Care to Cancer Patients: A Scoping Review

**DOI:** 10.3390/curroncol32060337

**Published:** 2025-06-07

**Authors:** Omar Alqaisi, Maha Subih, Kurian Joseph, Edward Yu, Patricia Tai

**Affiliations:** 1 Faculty of Nursing, Al-Zaytoonah University of Jordan, Amman 11733, Jordan; maha.subih@zuj.edu.jo; 2 Cross Cancer Center, Department of Oncology, University of Alberta, Edmonton, AB T6G 2R3, Canada; kurian.joseph@albertahealthservices.ca; 3 Department of Oncology, Western University, London, ON N6A 3K7, Canada; eyu@uwo.ca; 4 Department of Oncology, University of Saskatchewan, Saskatoon, SK S7N 5A2, Canada; ptai2@yahoo.com; 5 UpToDate, Waltham, MA 02451, USA

**Keywords:** nursing, attitudes, knowledge, practices, cancer patients, sexual care

## Abstract

Sexual health in cancer care is often overlooked. This study examines oncology nurses’ knowledge and practices regarding sexuality care, identifying barriers and facilitators. A Preferred Reporting Items for Systematic reviews and Meta-Analyses (PRISMA)-guided search of Scopus, ScienceDirect, PubMed, and EBSCO focused on studies from 2014 to 2024. Of 1735 identified studies, only 11 met inclusion criteria. Findings revealed a lack of knowledge among nurses and dissatisfaction with sexual healthcare. Barriers include time constraints, cultural factors, and personal reservations. Routine discussions are often absent due to inadequate training. Education- and system-based strategies are needed to enhance nurses’ competence in addressing sexual concerns. Implementing training programs, structured records, evaluation tools, concept maps, and system support would improve patient care and oncology nursing practices. Addressing these gaps with practical measures can enhance communication, patient satisfaction, and quality of life. This unique analysis was conducted by two experienced advanced nurses in the Middle East, where discussions about sex are often regarded as taboo.

## 1. Introduction

Cancer is the second leading cause of death worldwide, according to the World Health Organization. In 2022, an estimated 20 million new cancer cases and 9.7 million deaths occurred. Based on currently available data from internet searches at the time of writing this manuscript in May 2025, cancer accounts for approximately one in six deaths worldwide [1,2]. The leading types of cancer worldwide were lung, colorectal, liver, and stomach cancers in 2023 [2,3]. Among men, the most common types of cancer are lung, prostate, colorectal, stomach, and liver cancer. In women, the most prevalent cancers are breast, colorectal, lung, cervical, and thyroid cancer [4,5].

Sexual health is a fundamental aspect of overall well-being, influencing quality of life across physical, psychological, and social dimensions [6]. According to the World Health Organization (WHO) (2009), sexual health is an integral component of well-being [7]. Cancer can disrupt daily activities, particularly sexual functioning, as the disease and its treatments may lead to loss of sexual desire, poor body image, and impaired sexual performance [8]. Cancer and its treatment have been linked to sexual dysfunction [9]. Male partners may experience erectile dysfunction, while females may face vaginal dryness and pain during sexual activity [10,11].

Both patients and their partners may be uncertain or hesitant about addressing sexual concerns, as cultural taboos often discourage discussions about sexuality in the context of illness [12]. Despite advancements in cancer chemotherapy and radiation therapy, patients undergoing these treatments may experience irreversible sexual dysfunction [13,14]. Therefore, open discussions with healthcare providers about sexual care are essential [8]. Additionally, providing social support plays a crucial role in helping patients cope [15].

Sexual health information can help reduce anxiety related to sexual concerns, enhance sexual satisfaction, and improve patients’ overall quality of life [16]. Akhu-Zaheya et al. found that education on sexual health may help lower anxiety levels in patients [13]. Schwarz et al. noted that sexual health in cancer care is rarely prioritized, primarily due to language barriers, healthcare workers’ lack of knowledge on sexual health issues [17], and cultural and traditional factors that influence nurses’ perceptions of sexual care [14].

Understanding nurses’ attitudes toward sexual health, along with providing adequate training, is crucial for improving cancer treatment [18]. Cancer patients have sexual health needs, and unaddressed sexual dysfunction significantly reduces satisfaction, regardless of age, sex, or cancer type [19].

This scoping review explores existing literature on nurses’ attitudes, knowledge, and practices regarding sexual care for cancer patients. It identifies key factors influencing care delivery, including barriers and facilitators in different countries. By mapping current evidence, this review provides insights into how healthcare providers can better support the sexual health of cancer patients, ultimately enhancing care quality and patient outcomes. We aim to improve sexual care for cancer patients globally, including in cultures where discussing sex is considered taboo.

## 2. Materials and Methods

A comprehensive search was conducted across four major electronic databases: Scopus, ScienceDirect, PubMed, and the Elton B. Stephens Company (EBSCO) database, including Medline and CINAHL, focusing on peer-reviewed studies indexed in Web of Science and Scopus [20]. EBSCO is provided by the university for free, without any conflict of interest.

The search utilized a combination of keywords, including ‘nurses’, ‘attitudes’, ‘knowledge’, ‘cancer patients’ or ‘oncology patients’, and ‘sexual health’ or ‘sexual care’. The Mixed Methods Appraisal Tool (MMAT) was used as a checklist to simultaneously appraise and/or describe studies [21]. We used the Preferred Reporting Items for Systematic Reviews and Meta-Analyses (PRISMA) methodology to analyze past publications [22].

### Inclusion and Exclusion Criteria

The review included studies that met the following criteria: qualitative, quantitative, or mixed-methods research on nurses providing care to male or female cancer patients across various healthcare settings, published in English between 2014 and 2024. Data extraction followed a standardized form, capturing information on authorship (year of publication), study design, and results (knowledge, attitudes, practices, barriers, and facilitators). The data were extracted and evaluated by the first two coauthors (O.A., M.S.), while all coauthors critiqued the results for clinical relevance.

## 3. Results

**Figure 1:**
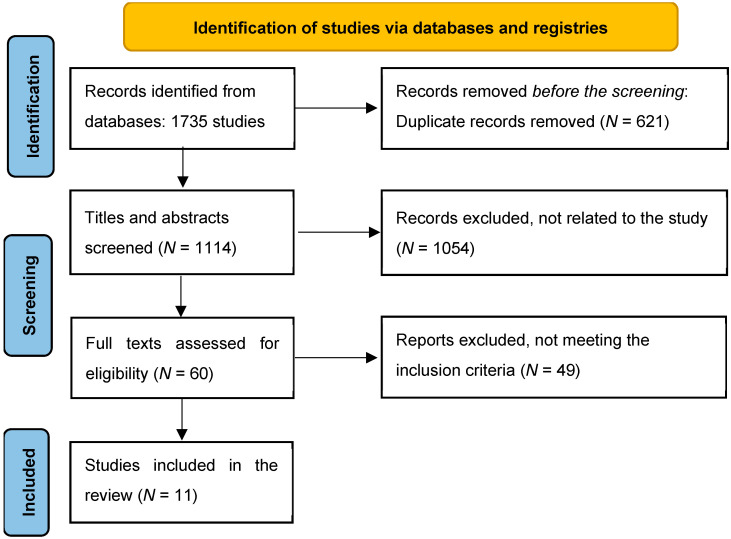
Flow diagram.

The database search identified 1735 studies across the selected platforms. These were reviewed, duplicates were removed, and exclusions were made based on the study inclusion criteria. Titles and abstracts were assessed, resulting in the selection of studies eligible for full-text review to determine their final inclusion. The search process is outlined in the following flow diagram (Figure 1). The qualifying studies were analyzed to examine nurses’ knowledge, attitudes, and practices in delivering sexual care to cancer patients. A total of 11 papers were included in the final review, comprising quantitative and qualitative survey studies, quasi-experimental interventions and concept mapping studies.

### Summary of Key Findings from Selected Studies

A significant finding across almost all studies is the widespread lack of knowledge among nurses regarding sexual care [17,23]. While nurses generally recognize the importance of addressing sexual health in cancer care, various barriers commonly hinder them from doing so:

1. *Discomfort in discussing sexual health* is a major challenge. Among Belgian oncology nurses, 85% reported feeling embarrassed when discussing sexual dysfunction with patients [24]. Similarly, in the study by Depke and Onitilo, 70% of nurses expressed the same concern [25].
2. *Lack of time:* In one study, 70% of Turkish nurses cited time constraints as a limiting factor when discussing sexual health [26]. Likewise, 60% of nurses participating in the Fex-Talk study reported similar challenges [27].
3. *Alignment with cultural sensitivities:* Chow et al. in 2021 used concept mapping to highlight the need for culturally appropriate care for the Chinese population, emphasizing that any developed model should *align* with the local clinical practice environment [28]. A three-phase concept mapping approach was adopted. Phase I involved individual interviews, Phase II generated a concept map, and Phase III evaluated its clinical applicability [28]. A total of 80 participants, including patients with gynecological cancers, their spouses/partners, nurses, and physicians, were recruited from two hospitals in Hong Kong. In Phase I, 50 statements were identified. Phase II used statistical techniques to create a concept map with seven clusters, including treatment impact, organizational support and information-giving. Phase III informed the development of an adapted practice model, based on the extended Permission, Limited Information, Specific Suggestions, Intensive Therapy (PLISSIT) model, which the concept map helped to shape [29]. Participants found this model appropriate for guiding sexuality care delivery [28]. Consistent with the above, Oskay et al. found that 60% of nurses in Turkey felt bound by cultural ethical values that prohibited discussions about sexual topics [26].

Practice tips evolving from our current study identify several facilitators that could help nurses provide better sexuality care:

1. *Educational interventions*: For instance, Winterling et al. found that 75% of nurses reported increased confidence following the intervention [27]. According to findings by Eid et al. and Winterling et al., structured training, practice, and workshops—particularly those incorporating focused role-playing—enhanced nurses’ self-perceived preparedness for discussing sexual health [27,30].
2. *Structured tools and guidelines*: Jung and Kim’s study highlighted a frequently overlooked issue: the importance of utilizing sexual healthcare (SHC) records for cancer patients to enhance nursing practice related to sexuality concerns [31].
3. *A nursing chronicle focused on SHC* for cancer patients may help facilitate and improve oncology nurses’ efficiency in delivering this type of care to their patients [32]. The authors emphasized the need for guidelines to standardize psychosexual care.
4. *Supportive environments*: The importance of the healthcare environment and team-based approaches was also highlighted. Paulsen et al. and Williams et al. affirmed nurses’ perspectives, stating that favorable systems and strong nurse–patient relationships encouraged greater disclosure [32,33]. Extracted summary is shown in Table 1.

## 4. Discussion

This review aimed to explore the existing literature on nurses’ knowledge, attitudes, and practices regarding sexual care for cancer patients. Three studies highlighted a significant knowledge gap among nurses (Table 1). Although they recognize the importance of sexual healthcare, factors such as limited understanding, embarrassment, time constraints and cultural barriers hinder effective communication in this area.

Despite widespread discomfort and lack of awareness, nurses acknowledge the significance of sexual health in cancer care. However, many report feeling embarrassed when discussing sexual health concerns with patients [25]. Attitudes are significantly influenced by cultural factors. For instance, Turkish nurses often perceive cultural conventions as barriers to discussing sexual matters [26]. Despite being aware of the importance of sexual health, nurses often refrain from actively engaging in discussions due to time constraints, lack of protocols, and inadequate skills. Educational methods, such as structured training and role-playing, can enhance their readiness and confidence [27]. Structured tools and criteria improve communication and attitudes [31]. Additionally, a supportive environment fosters honest and open conversations [32,33].

Table 1 supports several previous studies in the existing literature by addressing knowledge gaps, providing culturally appropriate solutions, and establishing a framework for enhancing sexual healthcare in cancer nursing practice worldwide [25,26,27,28,29,30,31,32,33,34]. The relevant references are discussed below. A published work from our university illustrates the cultural taboo surrounding discussions of male sexual impotence [35]. This review significantly contributes to the field.

Kotronoulas et al. concluded that nurses’ knowledge, attitudes, and practices in sexual and reproductive healthcare were inadequate [36]. Consistent with recent findings from Winterling et al. in 2020 and Paulsen et al. in 2023, interviewed nurses identified their educational deficits and insufficient training as key obstacles to addressing sexual health with cancer patients [27,33]. This highlights a persistent need for enhanced education and structured interventions to improve nurses’ communication skills in this area. Lack of education not only affects nurses’ knowledge but also impacts their confidence in addressing these issues. Despite efforts to advance nursing practices that aim to reduce sexual health inequalities, many nurses feel underprepared due to insufficient training.

Cultural factors also influence nurses’ attitudes toward sexual care. For instance, in studies conducted by Zeng et al., Chinese nurses reported that cultural norms posed significant challenges and served as reminders for oncology patients regarding sexual health [37,38]. Oskay et al. also highlighted that Turkish nurses faced cultural barriers in addressing sexual concerns. Both individual and societal factors prevented them from providing adequate support, despite the importance of sexual healthcare [26]. Discomfort in discussing sexual topics is often culturally rooted, particularly in conservative societies where sexual health remains a taboo subject. Cultural norms shape both nurse and patient perceptions, making it difficult to integrate sexual care into routine oncology practice. There are clear links between embarrassment, societal pressure, and the tendency of many Turkish nurses to avoid addressing sexually related matters [39]. Similarly, the current review identified cultural sensitivity as a significant barrier.

According to Kim et al. and Arikan et al., improving nurses’ attitudes and beliefs toward sexual care requires targeted interventions, such as training programs and the development of sexual healthcare scales to assess nurses’ readiness [40,41]. These observations are consistent with Jung and Kim in 2016, who suggested that the use of several specific aspects of a formal sexual healthcare nursing note would enhance nurses’ attitudes [31]. Structured interventions help create an organized pattern for addressing sexual health concerns, thus helping nurses navigate these discussions without experiencing the embarrassment often reported [31]. Interventional programs provide a structured approach, enabling nurses to address sexual health more systematically, thus reducing personal discomfort and facilitating better patient care.

There are different research techniques, one of which is the mixed method research (MMR) [42]. Table 1 summarizes different practical methods in the literature to enhance sexual care of patients. Methods for sexual counseling based on PLISSIT (Permission, Limited Information, Specific Suggestion, Intensive Therapy) and BETTER (Bring up, Explain, Tell, Time, Education, Record) should be in the nursing curriculum [43]. Different tools are available nowadays including scales for measurement [44]. Since cancer patients experience various post-treatment complications, research aimed at improving all aspects of quality of life—especially sexual health—is urgently needed.

Similar to nursing education, the core medical school curriculum does not adequately prepare students to discuss sexual health issues in their future careers [45,46]. These responsibilities often fall to coronary care nurses, especially when caring for patients recovering from a heart attack [47]. Sexual health is a crucial aspect of patients’ quality of life. In the minds of family doctors, they may feel incompetent to address all aspects of health for women of reproductive age [48]. Therefore, this study has broad *implications* for nursing, medical, and other health professional training programs, as well as ongoing continuing education after graduation. Obviously, breast, gynecologic, prostate, penile, testicular cancer patients would benefit from this study [49,50]. Other cancer types may also lead to post-treatment sexual morbidity. This study could serve as an important resource not only for cancer professionals but also for those involved in pre-exposure prophylaxis for Human Immunodeficiency Virus (HIV) transmission [51], sex education of teenagers and university students [52], and health surveys [53] aiming at improving communication and counseling.

Future research employing creative techniques may be beneficial, such as group sessions, phone conversations [54], artificial intelligence chatbots (especially useful when human resources are limited) [55], and protected online communities [56]. Research should also focus on specific subgroups of patients, such as sexual minorities [57,58], as well as individuals with special health conditions, including cardiac diseases, neurological disorders [59], or cystic fibrosis [60], etc. Their sexual health is often overlooked.

There are limitations to the present review. First, a large proportion of the reviewed studies relied on self-reported data, which carries a risk of bias. Nurses may have overestimated or underestimated their comfort level in handling sexual health-related duties. Additionally, potential language bias may exist, as only English-language articles were included. Furthermore, the diversity of healthcare systems represented across studies may affect generalizability.

In summary, this concise review, supported by valuable references, highlights a clinically significant issue that has received limited attention in publications. Notably, this work has been prepared by experienced and highly educated advanced nurses and researchers, whose backgrounds in ancient cultures have been shaped by religious beliefs that historically rendered discussions about sex with patients a taboo topic [35]. We are confident that both medical, nursing, and other healthcare professionals will benefit from adopting these suggestions. This work will serve as a valuable resource for all healthcare providers and educators.

## 5. Conclusions

This scoping review highlights the need for education, systematic reinforcement, and cultural competence to enhance oncology nurses’ ability to address the sexual health concerns of cancer patients. Despite perceived barriers, research has demonstrated that educational sex-related awareness sessions and structured interventions help ensure effective communication. 

Further research should be encouraged to quantify these interventions over the long term and assess strategies for integrating sexual medicine into broader oncology practice across diverse cultural and healthcare settings. If these challenges are managed effectively, healthcare providers will be better positioned to deliver quality care that acknowledges the sexual health needs of cancer patients, thereby enhancing their quality of life. 

Implications for practice are as follows: (1) To improve oncology nurses’ knowledge of sexual care for cancer patients, this review suggests integrating comprehensive sexual health education into established nursing and medical school curricula and continuing education programs, with an emphasis on multicultural competence. (2) Ongoing feedback and research on the long-term effects of these interventions are also crucial in modern healthcare practice to evaluate their benefits in various clinical settings.

## Figures and Tables

**Table 1:** Studies included in this review.

References	Study Design	Sample	Main Findings	Barriers/Facilitators [as Identified by % of Respondents]
2020, Winterling J [27]	Quantitative/descriptive cross- sectional	Oncology nurses (*N* = 60) in Sweden	Short educational intervention increased readiness to discuss fertility and sexuality (75%); improved communication confidence scores by 30%.	Barriers: time constraints (60%); lack of training (40%). Facilitators: 85% found the short, structured program beneficial.
2023, Paulsen A [33]	Qualitative	Nurses in gynecologic cancer follow-up (*N* = 15) clinics in Kristiansand, Norway	Expressed discomfort (70%); unprepared to initiate sexual health conversations (80%); regularly addressed sexual health (only 20%).	Barriers: discomfort (70%); lack of training (80%); perceived patient reluctance (60%). Facilitators: familiarity with patients (40%).
2015, Depke JL [25]	Descriptive cross- sectional	Oncology nurses (*N* = 45) in Weston, Wisconsin, United States	Recognized the importance of sexual health (80%); actively assessing patients (22%); embarrassment (70%); assumed patient disinterest (60%).	Barriers: embarrassment (70%); assumed patient disinterest (60%). Facilitators: 50% believed training could improve discussions.
2014, Oskay Ü [26]	Descriptive cross- sectional	Oncology nurses in Turkey (*N* = 112)	Believed sexuality is important in care (65%); initiated patient discussions (15%); time limitations (70%); cultural barriers (60%).	Barriers: time constraints (70%); cultural sensitivities (60%). Facilitators: more training on culturally sensitive care (55%).
2016, Jung D [31]	Quasi- experimental	Oncology nurses (*N* = 38) in Korea	Structured records improved attitudes (80%) and increased comfort in discussing sexual health (75%).	Barriers: initial reluctance to adopt new records (30%). Facilitators: structured documentation helpful (80%).
2015, Mansour SE [34]	Mixed- methods approach	Oncology nurses in Egypt (*N* = 72)	Poor knowledge scores (mean: 7.3 ± 2.5 among all nurses); discomfort in discussing sexual health was strongly correlated with lack of knowledge (*p* < 0.05).	Barriers: limited resources, staff shortages and patient embarrassment. Facilitators: nurse–patient relationship, private setting, communication skills.
2021, Chow KM [28]	Concept mapping	Nurses, patients, and spouses (*N* = 80) in China	Developed a practice model based on 7 clusters, including attitude toward sexual care and timing of delivery. Concept map adapted from PLISSIT model.	Barriers: lack of organizational support and structured protocols. Facilitators: organizational support, structured care model.
2020, Mbalè E [24]	Qualitative	Oncology nurses in Belgium (*N* = 20)	Sexual dysfunction is under-valued: not discussing sexual health due to embarrassment (85%), lack of evaluation tools (65%).	Barriers: embarrassment (85%), lack of tools (65%). Facilitators: increased awareness and knowledge.
2020, Eid K [30]	Pre- and post-intervention	Oncology nurses (*N* = 65) in United states	Knowledge scores improved significantly post-workshop; barriers to discussing sexuality reduced by 25% at 3 months and sustained at 6 months.	Barriers: lack of knowledge. Facilitators: workshop improved knowledge, role-playing activities.
2017, Williams NF [32]	Qualitative	Nurses in Australia (*N* = 17)	Five themes emerged, including the influence of personal and professional experience on psychosexual care. Lacked system support (60%).	Barriers: lack of system support (60%), personal discomfort. Facilitators: guidelines, teamwork, experience-based confidence.

PLISSIT: Permission Limited Information Specific Suggestions Intensive Therapy.

## Data Availability

We have provided details regarding where data supporting reported results can be found, including links to publicly archived datasets analyzed or generated during the study.

## Abbreviations

The following abbreviations are used in this manuscript:

BETTER	Bring up, Explain, Tell, Time, Education, Record
EBSCO	Elton B. Stephens Company
MMAT	Mixed Methods Appraisal Tool (MMAT)
MMR	Mixed methods research
PLISSIT	Permission Limited Information Specific Suggestions Intensive Therapy
PRISMA	Preferred Reporting Items for Systematic reviews and Meta-Analyses
SHC	Sexual Healthcare
WHO	World Health organization
